# Lessons from COVID-19 for GCR governance: a research agenda

**DOI:** 10.12688/f1000research.111331.1

**Published:** 2022-05-12

**Authors:** Jochem Rietveld, Tom Hobson, Shahar Avin, Lalitha Sundaram, Lara Mani

**Affiliations:** 1 Centre for the Study of Existential Risk, University of Cambridge, Cambridge, UK

**Keywords:** COVID-19, Corona, pandemic, pandemic response, global catastrophic risks, GCR, counterfactual analysis

## Abstract

The Lessons from Covid-19 Research Agenda offers a structure to study the COVID-19 pandemic and the pandemic response from a Global Catastrophic Risk (GCR) perspective. The agenda sets out the aims of our study, which is to investigate the key decisions and actions (or failures to decide or to act) that significantly altered the course of the pandemic, with the aim of improving disaster preparedness and response in the future. It also asks how we can transfer these lessons to other areas of (potential) global catastrophic risk management such as extreme climate change, radical loss of biodiversity and the governance of extreme risks posed by new technologies.

Our study aims to identify key moments- ‘inflection points’- that significantly shaped the catastrophic trajectory of COVID-19. To that end this Research Agenda has identified four broad clusters where such inflection points are likely to exist: pandemic preparedness, early action, vaccines and non-pharmaceutical interventions. The aim is to drill down into each of these clusters to ascertain whether and how the course of the pandemic might have gone differently, both at the national and the global level, using counterfactual analysis. Four aspects are used to assess candidate inflection points within each cluster: 1. the information available at the time; 2. the decision-making processes used; 3. the capacity and ability to implement different courses of action, and 4. the communication of information and decisions to different publics. The Research Agenda identifies crucial questions in each cluster for all four aspects that should enable the identification of the key lessons from COVID-19 and the pandemic response.

## Introduction: the COVID-19 pandemic and preparing for future GCRs

As of March 2022, the coronavirus disease 2019 (COVID-19) pandemic has resulted in the registered deaths of at least 6 million people ( McPhillips 2022), though the actual death toll may be higher still, close to 17 million, according to the Economist ( The Economist 2021). ^1^ The pandemic has caused disruption to global supply chains, hampered humanitarian responses and hindered healthcare provision. Across the globe, it has impacted billions of lives and, in many instances, exposed inadequacies in societies, processes and institutions. The pandemic presents a grim set of lessons about how we handle global threats that, as humanity, we should learn, internalise and implement.

The ‘COVID-19 Lessons’ project at the Centre for the Study of Existential Risk looks into the lessons that can be learned from the COVID-19 pandemic and from the global response, specifically with a focus on improving our ability to handle future Global Catastrophic Risks (GCRs). It focuses on identifying and investigating the most impactful decisions and actions (or failures to decide or to act) that significantly altered the course of the pandemic, with the aim of improving disaster preparedness and response in the future. It also asks how we can transfer these lessons to other areas of risk management to help us address global challenges of potential catastrophic impact such as future pandemics, extreme climate change, radical loss of biodiversity and the governance of extreme risks posed by novel technologies.

There is, at present, no agreed definition of a GCR, with most definitions relating to global death tolls in the millions or billions ( Bostrom and Ćirković 2011, Avin *et al.* 2018, Turchin and Denkenberger 2018) and some looking further to catastrophic impacts in terms of economic loss or suffering ( *e.g.* Blong 2021). Depending on the definition used, COVID-19’s impact is either too small to count as a GCR, or just large enough to count on the smaller end of the scale, with the attention of the field still dedicated to scenarios that kill hundreds of millions or billions of lives. Nonetheless, the (fortunate) rarity of GCR events means GCR scholarship relies on smaller catastrophes for empirical evidence to understand higher-impact scenarios. The COVID-19 pandemic and, especially, the global response to it, now represents one of the most relevant sources of information for the study of GCRs. It bears more structural similarities to the GCR scenarios that humanity is likely to face in coming decades than with historic pandemics ( *e.g.* the 1918 influenza pandemic or the black death), past large-scale industrial disasters ( *e.g.* Fukushima or Chernobyl), or large-scale natural disasters ( *e.g.* the Toba eruption). While the specific mechanisms by which the pandemic spread globally and killed are familiar, the failures to prevent or mitigate it are numerous and highly relevant to GCR research and policy.

It should be noted that, among the range of potential GCR scenarios that humanity currently faces, pandemics are conspicuous because of how well prepared for them we are - at least in principle. We already possess a complex of national and international organisations dedicated to monitoring and responding to health threats, and there are existing infrastructures for research and development, along with robust pathways for investment. Healthcare spending and the infrastructural capacity and resilience of national health services do vary greatly by region and country, yet taken overall, the funding, knowledge and infrastructure that can be brought to bear on a pandemic outbreak significantly exceeds that which is presently available for addressing alternate GCR scenarios, such as environmental protection or asteroid deflection.

The crucial question is how we can ensure that the lessons from COVID-19 not only reach the desks of policymakers, but actually leads to lasting and effective policy and cultural change: that we come out of this pandemic safer and more resilient against a wide range of global risks, and that these lessons guide the actions of present and future generations. Global catastrophes of this magnitude are, fortunately, rare, but so are the windows of opportunity to truly change humanity’s attitude towards them.

### Phase one: understanding what happened and asking how it could have been different

Before we can draw lessons that are relevant for prevention and mitigation across a wide range of GCRs, it stands to reason that we first need to understand the decisions and actions that were instrumental in making COVID-19 a global disaster. Crucially, it is also necessary to gain some understanding of how existing (or novel) knowledge and governance mechanisms or changes in decision making could have plausibly prevented this outcome.

Efforts to understand different elements of the COVID-19 pandemic are already well underway. Governments, public health experts and planners are attempting to understand topics as diverse as the origins of the severe acute respiratory syndrome coronavirus 2 (SARS-CoV-2) pathogen (the virus that causes COVID-19) and the effectiveness of social distancing measures and other non-pharmaceutical interventions. This project’s focus is on understanding what the COVID-19 pandemic tells us about the nature of global-scale, systemic risks and to draw out the lessons that can be learned about how to better prepare for such risks in the future.

The project explores the ways that the pandemic could have gone differently. Through a detailed review of secondary literature and a series of expert interviews, we are working to identify key decision and action junctures, or “inflection points”, in the history (and prehistory) of COVID-19 to ask:

1. What were the decisions and actions that shaped the course of the pandemic? Who were the decision makers and in what context were they operating?
2. Were these particular decisions or actions inevitable? What other options were available?
3. If things could have been different, how? What would have been the (likely) outcomes of choosing or acting differently?
4. What changes in policy, preparedness or implementation would have made it possible for different, and better, responses to this type of global risk?

These *counterfactuals* make it possible to compare the actual course of the pandemic with *possible* and perhaps *better* courses.

Counterfactuals are “contrary to facts”, *i.e.* conditionals that identify a “possible” or “alternative” world in which the antecedent did not actually occur ( Levy 2008). They are useful in that they allow us to think of a world where different decisions—such as different public health interventions—were adopted. This method is not without its difficulties, as “we cannot rerun history” ( Sato 2021, p65). There are, however, various ways to increase the validity of counterfactual analysis, such as through maximising clarity and plausibility of the conducted analysis, which is what we are aiming for. Utilising both quantitative analysis on the basis of forecasting—and qualitative analysis—on the basis of expert elicitation and the analysis of documents—our research will explore what impact different decisions *could* have made in terms of lives lost and the humanitarian and social costs of the pandemic. These insights can provide vital lessons for decision-makers, now and in the future.

In our research to date, we have identified four broad types of issues that we believe had the most significant impact on the course of the pandemic, and which largely determined the observed death tolls. These four clusters are *Pandemic Preparedness, Early Action, Vaccines* and *Non-Pharmaceutical Interventions.*

The clusters are each structured as follows: (1) they assess what information was available to experts and decision-makers relevant to each cluster when COVID-19 broke out; (2) they assess decision-making processes, analysing what decisions were made, when, and how and if other decisions, potentially at different points in time, could have resulted in better outcomes in terms of the pandemic trajectory; (3) they assess implementation and infrastructure, *i.e.* what infrastructure and capacities relevant to the clusters at hand were available? Was this infrastructure up to the task to effectively address the pandemic? Regarding implementation, were decisions taken relevant to the particular clusters adequately implemented? If not, why was implementation lacking and how can this be improved in future responses? Where was there room for implementation improvement and how would this have potentially positively altered the trajectory of the pandemic? Finally, (4) they assess communication aspects relevant to the different clusters, as communication is key in navigating any crisis. For each of these four aspects, we indicate our current knowledge base and invite interested parties to share their insights with us.

Below, we provide a short overview of each cluster, giving some illustrative examples of each and offering some initial suggestions as to how *counterfactually* these issues or events could have been responded to differently, and the impact this could have had.

Most importantly, each cluster concludes with a set of “open questions''. These questions are efforts to bring together what we *do know* with an agenda for further research and a call for collaboration with other interested parties. We believe that working together to answer these questions can play a major part in supporting lasting and effective policy and cultural change so that we come out of this pandemic safer and more resilient against a wide range of global risks, and that these lessons are passed on to present and future generations.

While this document lays out an agenda for what we envision to be crucially important research to attain these goals, we also intend that it might form a useful reference document to a range of different readers: providing an overview of current knowledge relating to the COVID-19 pandemic and international responses to it.

## Cluster #1: pandemic preparedness

### Background

The first cluster of our research agenda looks into pandemic preparedness. The World Health Organization (WHO) defines pandemic preparedness as “a continuous process of planning, exercising, revising and translating into action national and sub-national pandemic preparedness and response plans” ( WHO 2022a). The organisation sees pandemic preparedness as an integral part of preparedness to threats to human health caused by any emergency, which includes disease outbreaks, but also occurrences of natural disasters or chemical incidents ( WHO 2022a). As pandemic preparedness is a continuous process, this makes pandemic plans living documents, which are to be “reviewed regularly and revised if necessary, for example based on the lessons learnt from outbreaks or a pandemic, or from a simulation exercise” ( WHO 2022a). This cluster asks what role pandemic preparedness had to play in the period leading up to the COVID-19 pandemic, where preparedness shortcomings emerged, as well as how notions of preparedness have started to evolve since the outbreak.

### Information

1. What information was available to policy makers and experts about preparations they should make for future pandemics? What information was available about the likelihood and severity of future pandemics?
2. What information did pandemic exercises deliver? Was this information followed up on?
3. What information was available regarding different pandemic preparedness options ( *e.g.* tabletop simulations, large-scale exercises, stockpiles, surveillance, *etc.*)? What information was available about the likely cost and efficacy of these options? What information was available about how best to utilise such tools?
4. What was the size and structure of the research community focusing on understanding and preparing for future pandemics? What were its key findings and focus areas for research? What were its pathways to deliver findings and recommendations to policy makers?

Although the knowledge base surrounding pandemics and pandemic preparedness was substantial ( Madhav *et al*. 2017), it can be argued that decision makers did not do enough with this information in preparing their countries for the eventuality of a pandemic. Part of the problem is that not all countries seem to have conducted pandemic preparedness exercises, and that those who did, did not always prepare for the “right” type of pandemic (there was a general bias towards preparing for pandemic influenza). It has also been noted that some countries organised their exercises in ways that were so theoretical in nature that they turned out to have little bearing on the outbreak of an actual pandemic. This was laid painfully bare by COVID-19 ( Salden 2020).

In addition to the knowledge that could have been garnered from pandemic exercises, there was a wealth of information on preparedness that had been accumulated from responses to previous outbreaks, such as those of severe acute respiratory syndrome (SARS), H1N1, Middle East respiratory syndrome (MERS) and Ebola. These outbreaks were invariably evaluated by high level panels, which then produced reports providing decision-makers with detailed information on what measures they should take to be better prepared for the next pandemic ( IISD 2016). Our initial research suggests that decision makers in government and elsewhere frequently acted inadequately in response to this type of information and that this contributed to a lack of preparedness when SARS-CoV-2 was discovered in late 2019.

Finally, while many countries had developed pandemic preparedness plans in recent decades, there are a number of ways that the effectiveness and adequacy of these plans might be questioned. For instance, following the 2009 H1N1 pandemic, many European countries revised their national pandemic plans to prepare for future influenza pandemics and to strengthen implementation of the International Health Regulations (IHR 2005) ( WHO 2022b). The revised plans considered the national and global experiences from the 2009 pandemic, and in some countries, they were used to form the basis of the initial national response to COVID-19 ( UK Department of Health & Social Care 2020). Yet, some of these plans cautioned against measures that have become commonplace in the COVID-19 pandemic response, such as mask-wearing and border closures ( Blanco-Jimenez 2021). We suggest that it is important to understand the causes of this disparity and investigate national and regional variations in planning.

**Our current knowledge base on this information-aspect is moderate, so we invite people who could provide further insights into the role of information in pandemic preparedness to share their insights with us.**

### Decision making

1. Did decision makers decide to organise pandemic exercises? If so, why and how? Why did they run them in particular ways? Who participated in such exercises, and who did not?
2. Was information resulting from pandemic exercises followed up on? If yes, how? If not, why not?
3. What other pandemic preparedness actions were considered? Why were they pursued, or not pursued, in different countries at different times?

WHO ran a number of initiatives that sought to prepare member states for pandemic influenza, such as the Pandemic Influenza Preparedness (PIP) Framework (2011) though, notably, not for other types of pandemics ( WHO 2022c). Similarly, many countries ran pandemic scenario exercises in the years leading up to the COVID-19 outbreak; however, these were also largely focused on how to respond to pandemic influenza, and our initial research suggests that this may have been to the detriment of their preparedness for other types of viruses. Even instances where an influenza preparedness plan may have provided the right kind of guidance for the COVID-19 pandemic, the specific content of these plans and the actions that governments took based on their recommendations, should still be considered as important elements of the global pandemic response.

It is possible that these preparedness plans and exercises, focusing on influenza pandemics, may have framed the expectations of decision makers. Hence, officials may have been caught off guard by the outbreak of a coronavirus pandemic. In the case of the UK, it has now emerged that the government based its early response on pandemic influenza scenarios, even though exercises *had* been conducted on coronaviruses (Booth 2021a). In the early weeks of the pandemic, there was a considerable degree of uncertainty about the transmissibility and lethality of COVID-19 compared with influenza, which complicated assessments of the novel virus. Here, tapping into the knowledge delivered by conducted *coronavirus pandemic exercises*—as opposed to *pandemic influenza scenarios*—would have been appropriate and therefore constitutes a missed opportunity. By now, a consensus exists that all SARS-CoV-2 variants encountered by March 2022 are causing significantly more morbidity and mortality than recent influenza strains. It can be assumed that this is due to a combination of higher transmissibility and lethality leading to an overall larger burden of disease despite significantly higher lethality for example of some Highly Pathogenic Avian Influenza (HPAI) strains which have in recent decades not been able to transmit more than sporadically to humans ( Hammers 2022).

Another issue has been the disjuncture between the highly theoretical way in which some pandemic exercises were set up and the very real events and consequences of an actually occurring pandemic. A useful illustration of this is provided by the Netherlands, which, in 2019, organised an exercise to simulate the outbreak of an influenza epidemic. This exercise focused largely on communication and on coordinating management responsiveness within healthcare systems, leaving little room to consider some of the concrete dilemmas, policy trade-offs and pressures that a real pandemic would bring about.

Practitioners who participated in the simulation later reflected that the exercise was totally incomparable to the actual pandemic of COVID-19, and that at the time of the exercise they saw it as a merely theoretical exercise. One of them noted that a crisis exercise does not have a “face”, whereas the actual pandemic very much did: healthcare practitioners saw exhausted colleagues, overwhelmed hospital managers, and patients who were short on breath ( Salden 2020). One of the participants, a governor of a number of regional hospitals, reflected amidst the first COVID-19 wave in Spring 2020: the exercise was “very useful, but no one thought this [outbreak] was ever going to happen” ( Salden 2020). In the same vein, the international Independent Panel for Pandemic Preparedness & Response (2021, 20) (hereafter: the Independent Panel) also noted the need “for preparedness assessment to place more focus on the way the system functions in actual conditions of pandemic stress.” ^2^

As noted, information resulting from pandemic exercises was not always adequately followed up on. Preliminary information seems to suggest that the UK ran a large number of exercises but did not implement key recommendations from them, such as the need for PPE stockpiles and better contact tracing systems ( Booth 2021b). This may have been the result of a lack of political prioritisation, which appears to have been an issue in many countries. In the United States, for example, pandemic preparedness was made a priority under the Obama administrations, but under the Trump administration, the position of pandemic adviser was downgraded, and the relevant official was no longer able to convene the cabinet ( Tracy 2020). The impact of shifts in political prioritisation is also demonstrated by the decision of California’s administration, under the leadership of Brown, to sell off or destroy the stockpiles of ventilators and PPE that had been built up under the Schwarzenegger administration ( Marinucci 2020).

**Our current knowledge base on this aspect is high, but we do invite people who could provide further insights into the role of decision-making in pandemic preparedness to share their insights with us.**

### Infrastructure/implementation

1. What did the pandemic exercises look like? How were exercises structured and why?
2. Did pandemic exercises result in new infrastructure/capacities? (PPE, vaccine development capacities, investment in R&D). If yes, how? If not, why not?

Pandemic preparedness exercises were conducted in many countries ( WHO 2018), but many of these exercises seem to have inadequately prepared countries for a real pandemic, or at least, for the current coronavirus pandemic. An important part of the problem, as noted above, is that exercises were often inadequately followed up on, which means that they did not deliver significant new health capacities and infrastructure, such as stockpiles of PPE, vaccine development capabilities, scalable ICU capacity, and track and trace systems.

A related problem, that exacerbated the failure of many countries to act upon the lessons learned from their preparedness exercises, was the more generalised underfunding of pandemic preparedness. The Independent Panel (2021, p. 56) highlighted this lack of funding for pandemic response, while also noting that in some places, periods of adequate funding and capacity building were followed by budget cuts and capacity reduction. Whether these cycles were influenced by generalised issues of political prioritisation or by shifting attitudes towards the costs and benefits of maintaining large buffers of residual capacity for health emergencies, the consequences of the cuts appear stark in many countries. The aforementioned example of California demonstrates clearly how these reductions in capacity occurred in the years preceding the COVID-19 pandemic. The UK’s pandemic influenza stockpile is another example. It was estimated at GBP 831 million in 2013, but declined by 40% over six years ( Davies *et al*. 2020).

Preliminary evidence suggests that some countries were better prepared, and these countries tended to be those that had previous recent experience with large-scale outbreaks, such as Southeast Asian countries (with SARS) and West African countries (with Ebola) ( Ahanhanzo *et al.* 2020; Nuki *et al.* 2020). These countries acted swiftly in introducing measures to control and monitor the spread of SARS-CoV-2. In many cases, it appears that they had—as a result of their more recent experience of viral outbreaks—also developed a public health infrastructure that was better able to respond to the outbreak of a pandemic (Independent Panel 2021). It should be noted, however, that pandemics necessarily entail different phases and that countries’ early performance in the COVID-19 response is not necessarily a predictor of later performance in pandemic response ( Frieden 2021).

**Our current knowledge base on this aspect is limited, so we invite people who could provide further insights into the role of infrastructure/implementation in pandemic preparedness to share their insights with us.**

### Communication

1. Did decision-makers communicate the importance of preparedness? If so, how? If not, why not?
2. Did decision-makers aim to create awareness among the general public about pandemic risk and societal resilience against this risk? If so, how? If not, why not?

Public communication has an important role to play in pandemic preparedness, informing the public about the risk of pandemics and preparing it for eventual outbreaks. Information provision and communication with regards to pandemic risks could also run through the education system, creating basic health awareness among children, including teaching them the basic tools of staying safe during a pandemic. The Disaster Risk Reduction (DRR) literature has demonstrated the importance of the role of education in disaster preparedness ( Johnston *et al.* 1999, Ronan and Johnston 2003, Shaw *et al*. 2004, Paton *et al*. 2008). Preliminary information seems to suggest that there were generally few awareness raising campaigns from governments about the risk of pandemics and the importance of preparedness towards their publics. It is important to assess the extent to which societal and political awareness could have helped in the (early) response to COVID-19. If public communication and awareness turns out to be a significant aiding factor, the question becomes: how can we sustain pandemic communication and awareness after the current COVID-19 outbreak? Potential lessons can be learnt from countries with previous pandemic experience that built up pandemic public communication systems before the outbreak of COVID-19.

**Our current knowledge base on this aspect is high, but we invite people who could provide further insights into the role of communication in pandemic preparedness to share their insights with us.**

On a final note, it is important to acknowledge that “pandemic preparedness” has a very different meaning today compared to pre-Covid times. In the face of the very real COVID-19 pandemic, many more elements have now been added to the “preparedness toolbox”. We recognise how the term “pandemic preparedness” has shifted over time and are interested in evaluating the pandemic response through both understandings (pre- and post-Covid) of the term.

### Questions

The following is a (non-exhaustive) list of some of the key “open questions” that we have identified to date for this cluster of issues. These are indicative of the ongoing research agenda guiding this phase of our project, and we would be particularly keen to speak with practitioners and experts that can shed further light on answers to some or all of these questions:

1. What constitutes a successful pandemic plan and an effective pandemic preparedness training exercise?
2. How much funding is required to successfully realise these plans and exercises nationally and regionally? And how might this figure differ across states with different levels of wealth and different institutional capacities?
3. How can global, regional, and national actors work together to ensure that relevant capacity to achieve exercises is built across the globe and that key recommendations flowing from exercises are actually being implemented?

In other words, we aim to answer the following overarching questions:

***What kind of pandemic preparedness—in terms of knowledge, institutional capacity, resources, and training—would actually serve to make sure that the world was better prepared for the next pandemic?***

***What can we learn from the successes and failures of various efforts to prepare for a global pandemic that will allow us to better prepare for other categories of Global Catastrophic Risk, such as those associated with climate change, the loss of biodiversity, and the rise of potentially disruptive technologies?***

## Cluster #2: early action

### Background

This cluster focuses on the early action phase in the pandemic response. We define the “early action phase” as the initial months of the COVID-19 pandemic from December 2019 until the summer/early autumn of 2020. ^3^ This period encompasses the initial detection of the virus in China, early outbreaks on every inhabited continent, and the end of what came to be known as the “first wave” of infections, and the end of a first round of “lockdown” restrictions in many countries.

In the very early phases of disease outbreak, a rapid containment strategy may still be feasible. The WHO defines rapid containment as effectively “stopping the development of a pandemic when it is initially detected before the virus spreads more widely” ( WHO nd.). Once the virus spreads more widely, however—across countries and continents—countries are forced to respond to community level outbreaks and will usually consider a wide range of public health measures to contain, suppress, or eliminate these outbreaks. Our “early action phase” considers both of these phases.

### Information

1. What information was available about SARS-CoV-2 in the early days and weeks immediately following its discovery?
2. What assumptions were prevalent in the expert community about the virus and its transmissibility and lethality in the early days? How were these communicated to decision-makers?
3. What information was available about the effectiveness of various containment measures in the early days? What models were used, and how were they presented to decision-makers? What were the main arguments from experts against various forms of early containment?
4. What information-gathering approaches were used in the early days? What other approaches were available, and why were they not pursued?
5. What information-sharing infrastructure existed on the eve of the pandemic? What information-sharing infrastructure was stood up during the early days? Who was included and who was excluded from such networks?

Little was known about COVID-19 when the first few cases of the novel disease emerged. There was ongoing uncertainty in the first few weeks about the prevalence and precise mechanisms of human-to-human transmission and the extent of asymptomatic spread ( Parry 2020; WHO 2020).

Regarding the effectiveness of containment measures, decision-makers had to take clues from what was implemented—effectively or otherwise—in countries that experienced the earliest outbreaks, as well as from modelling of related diseases and historic knowledge concerning respiratory viruses and the spread of disease. There was, however, considerable uncertainty at the time, and there were competing interpretations and recommendations coming from a range of actors across the scientific community. It was against this background of uncertainty that decision makers had to chart a course of action and shape the early pandemic response.

**Our current knowledge base on this aspect is medium, so we invite people who could provide further insights into the role of information in early action to share their insights with us.**

### Decision making

1. Which containment measures did decision makers choose and how did they come to these decisions?

During any crisis, effective decision making in the early response is vital. Unfortunately, this element was not always present in the early response to the COVID-19 pandemic. While we acknowledge that decision making efficacy will always be easier to adjudge with the benefit of hindsight—and the limited information, higher uncertainty and the existing bounds of infrastructural capacity will shape decision-making processes—our research to date suggest a number of instances where robust decision making may have significantly altered the trajectory of the pandemic.

When considering the possibility of early containment for example, China could have banned commercial flights leaving the country earlier, while other countries could have closed their borders sooner. While these types of intervention may have seemed drastic or even draconian at the time, it is now clear that avoiding such decisions may have led to the longer-term imposition of both border closures and disruption to international travel.

It should also be queried why WHO did not apply the precautionary principle in its decision-making (or if it did, then what other considerations were factored in that took precedence). The precautionary principle entails assuming that human-to-human transmission is taking place in an outbreak of a novel pathogen unless evidence to the contrary emerges (Independent Panel 2021).

The costs of applying the precautionary principle would arguably have been far lower than the cost of choosing to delay action or prioritising the continuation of trade, travel or business; thus, insufficiently preparing for and mitigating against an outbreak that would prove to be as serious as the COVID-19 pandemic (Independent Panel 2021).

Despite this, China and other Asian countries did take early containment measures internally, including the imposition of lockdowns, the disinfection and shutting down of wet markets and bans on domestic travel ( Graham-Harrison 2020). Many other countries, including the majority of European states, tended to react slowly, adopting a wait-and-see approach and appearing rather unprepared for the COVID-19 pandemic ( Lawler 2020). This once again demonstrates the interaction and interdependence of robust decision making with various aspects of pandemic preparedness. On the one hand, gaps emerged in what processes were ready to be deployed, what institutional capacity existed, while at the same time, mistakes were made in implementing plans and setbacks occurred in deploying processes that had been prepared.

Besides individual country approaches, there was a lack of international leadership early on in the pandemic. The G7 and G20, United Nations (UN), and WHO all fell short in taking effective and timely measures and providing global and regional leadership at the time it was most needed ( Wilson & Pilling 2020; AlQershi 2020). Coordinated measures at the highest levels could have made it easier for countries to take tough measures themselves and explain these to their citizens. Unfortunately, global and regional measures either did not come, came late or were insufficient. Leadership rivalry and contradictory approaches carried the day, whereas cooperative leadership and joint efforts were mostly lacking ( Wright 2021; OECD 2020). It is understandable that there is a strong temptation for countries to adopt a “my country first”-approach during crises, but when it comes to global challenges with systemic effects, this appears to have been a self-defeating strategy in the medium-to-long term, not least in the face of a pathogen that spreads without consideration of national borders.

**Our current knowledge base on this aspect is medium, so we invite people who could provide further insights into the role of decision-making in the early response to share their insights with us.**

### Infrastructure/implementation

1. Did countries have the infrastructure/capacities to close borders, enforce lockdowns etc.?
2. Did countries have the capacity to support individuals in isolation?
3. Did countries have the capacity to enact targeted containment through testing and tracing?

When analysing the early response, it is important to assess whether countries possessed the relevant infrastructural capacity to implement public health decisions and containment measures. Did countries have the infrastructural capacity and organisational capabilities to close their borders, enforce lockdowns, and track and eliminate infection chains? Were there sufficient policy and legal capacities and clarity to enact such measures rapidly? Much has been reported about the challenges and dilemmas of low-income countries to implement lockdowns, given high dependence on daytime wages in the informal sector ( Piper 2020). Equally, not all countries have had the capacity (economic or indeed political) to support individuals in isolation, which is an important tool to curb infections.

**Our current knowledge base on this aspect is limited, so we invite people who could provide further insights into the role of infrastructure/implementation in the early response to share their insights with us.**

### Communication

1. How did decision-makers communicate early action measures to their publics?
2. What kind of communication approach was required in this early phase?

Finally, there were communication inconsistencies and mistakes early on in the pandemic. These occurred at all levels, but the frequency and severity of poor communication and mixed messaging from senior levels of the political executive (in a number of states) seems particularly important to note. A seemingly minor but very telling example is how some leaders accidentally shook hands with other officials after publicly announcing people should stop doing so themselves, while others even boasted about the practice, in clear defiance of warnings from the scientific community ( BBC 2020a; Mason 2020).

More serious examples came from, amongst others, Brazil and the USA. President Jair Bolsonaro directly contradicted the advice provided by the Brazilian Governments’ Health Ministry by urging the Brazilian public not to comply with social distancing and other public health measures ( Human Rights Watch 2020). In the USA, there was a considerable flow of misinformation emanating from the Trump administration with regards to the pandemic and measures to contain it ( Sauer *et al*. 2021). There were a number of examples of effective public communication by national leaders as well. Particularly notable examples include Germany’s Angela Merkel, New Zealand’s Jacinda Ardern and The Netherlands’ Mark Rutte, who communicated factually, cautiously and reassuringly in the early days of the pandemic ( Wilson 2020; Delahunty 2020; Brassey and Kruyt 2020). Singapore has also been commended for its effective and transparent communication strategy ( Sagar 2020).

Research has shown that reliable and speedy communication is crucial in navigating complex crises, such as pandemics. US researchers found that “communication should be rapid and accurate, while building credibility and trust and showcasing empathy—all with a unified voice” ( Sauer *et al*. 2021, p65). Public communication in the early months of the pandemic was especially important as it coincided with the rollout of early—and often unprecedented (at least in Europe/Northern America)—public health measures, such as social distancing, test and trace systems, and the prohibition of mass gatherings. The imposition of these measures, and ultimately their effectiveness, was largely dependent on public acceptance and cooperation. While most modelling for these so called non-pharmaceutical interventions clearly stated an acceptable degree of non-compliance within their efficacy predictions, effective public communication was required to successfully convince the public that they were necessary.

As much was still unknown about COVID-19 in the early pandemic, public communication necessarily included some discussion of uncertainty. According to Igoe (2021), there are two sources of uncertainty in science communication: uncertainty deriving from changes in knowledge, and uncertainty occurring when leaders and public figures contest or debate scientific findings in public ( Igoe 2021). It appears, from our research to date, that both categories of uncertainty were present in the early action phase of the pandemic response. Firstly, as new information became available, experts frequently had to revise and retract earlier public health advice or revisit their previous epidemiological models. At the same time, political leaders and public figures often sought to emphasise certain aspects of uncertainty, or to question particular scientific claims, in order to advocate for different political or economic prioritisations. Both these factors may have convoluted the clarity of public messaging, leading to public confusion at times in various countries ( Han *et al.* 2020). Where the uncertainty resulting from the rapidly shifting knowledge base concerning COVID-19 was not well-communicated, it may also have contributed to an undermining of trust in the scientists themselves.

Finally, alert level systems were introduced early on to communicate the risk of COVID-19. The UK unveiled a five-level, colour-coded alert system in May 2020, which ranks the current threat level from COVID-19 ( Sabbagh 2020). Colour-coded maps have also been widely used to accompany travel advice for different countries and regions ( European Centre for Disease Prevention and Control 2022).

**Our current knowledge base on this aspect is medium, so we invite people who could provide further insights into the role of communication in the early response to share their insights with us.**

### Questions

The following is a (non-exhaustive) list of some of the key “open questions” that we have identified to date for this cluster of issues. These are indicative of the ongoing research agenda guiding this phase of our project, and we would be particularly keen to speak with practitioners and experts that can shed further light on answers to some or all of these questions:

1. How can policy makers, practitioners and scientific advisers determine the most effective containment measures when much is unknown?
2. Why were borders not closed earlier and how much of a difference would this have made?
3. What is needed to achieve border closures and other effective containment measures as early as necessary during future pandemics? Why was coherent action and effective leadership lacking by the world’s global and regional institutions, such as the G7 and G20, the UN, WHO, the EU and other regional organisations?
4. How can public communication be improved in the next pandemic and/or other crises?

Again, we restate here that we are working towards answering two basic questions in relation to the early action phase:

***What kind of early responses—including containment, knowledge-sharing and communications—to the discovery of the COVID-19 outbreak would have significantly altered the trajectory of the pandemic, and significantly reduced the global loss of life, injury and harm?***

***What can we learn from the successes and failures of various early response efforts to contain or otherwise mitigate the COVID-19 pandemic to better prepare us to respond effectively to other categories of Global Catastrophic Risk; such as those associated with climate change, the loss of biodiversity, and the rise of potentially disruptive technologies?***

## Cluster #3: vaccines

### Background

Vaccines were developed at extraordinary speed and are likely to be remembered as one of a few success stories of the pandemic response. While 2020 saw the introduction of lockdowns, masks, and social distancing, to many, vaccines represented the only viable and sustainable way out of the COVID-19 crisis. Before the development of antiviral medicine such as molnupiravir and Paxlovid, it was believed that vaccines were the only medical means to protect people against the virus ( Aripaka 2021). Alongside non-pharmaceutical interventions such as social distancing *etc.*, a number of experts believed that mass vaccination programs would be able to, at least theoretically, end the pandemic. Current data regarding vaccine rollouts and the continual emergence of vaccine-evading variants, however, suggest this is no longer a realistic scenario ( Charumilind *et al.* 2021).

In the early months of the pandemic, multiple companies developed vaccines based on the genome sequence that was released in January 2020. The vaccines were developed at a remarkable speed and moved quickly through the various development, clinical trial, and authorization-for-use phases ( Ball 2020). The mRNA vaccines particularly received much attention, as this new generation of vaccines can be developed very quickly and thus proved very promising both from a scientific and public health point of view ( Ball 2020). In December 2020, the worldwide vaccination campaign truly started with the first people being vaccinated in the United Kingdom. Since then, billions of people have been vaccinated in a global vaccination campaign ( BBC 2020b; Bloomberg 2022).

For all the successes of the global effort to develop, manufacture and distribute vaccines for COVID-19, there do, however, remain a significant number of challenges and substantial gaps in the provision of these. The most significant among these remains the need to deliver and effectively distribute vaccinations to the populations of countries in the Global South. At the same time, even the vast scale of the vaccination programs rolled out in Europe and North America has failed to eliminate the spread of the virus; indeed, December and January of 2021/22 have borne witness to rising infection rates in a number of high-vaccination countries.

Commitments such as those made by the G7 to donate vaccines to the Global South are still in their implementation phase ( Reuters 2021; Gye 2021). Meanwhile, as of late September 2021, the WHO-led COVID-19 Vaccines Global Access (COVAX) was forecasting to deliver 1.45 billion vaccines through its scheme by the end of the year, compared with the 2 billion it forecast to deliver earlier in the year ( Horner 2021). The aforementioned tendency of some countries to prioritise purchasing additional vaccine doses for their own citizens, as opposed to distributing these to other countries has posed an important political challenge to vaccine equity around the world. Many countries in the Global North are now administering vaccine boosters (and indeed, even discussing an additional fourth booster dose) while many health care workers in the Global South are still waiting for their first dose ( Maxmen 2021). Another problem is mis- and disinformation surrounding vaccines, which has arguably led to a less successful vaccine rollout than desired ( Loomba *et al.* 2021).

A final challenge that has come to light is that vaccinations offer reduced protection against new variants of SARS-CoV-2, such as the Delta and Omicron variants, when compared to earlier strains of the virus. Still, many researchers hold that it is currently our best tool in the fight against the virus and the respiratory disease it causes ( Mancini and Burn-Murdoch 2021).

### Information

1. What did early prognoses of vaccine discovery/development look like?
2. How was information about vaccine efficacy and safety collected? How was it communicated to decision makers, medical professionals, and the public?
3. What information was available about the logistical challenge of “getting shots into arms”, and what solutions were offered by experts and by local practitioners? How was information about the successes and challenges of vaccine rollout fed back into further planning and rollout cycles?
4. Where does vaccine misinformation originate, how does it spread, and how much of an effect does it have on vaccine uptake? What actions were taken to counter vaccine misinformation, and were they effective?
5. What did proposals for rapid vaccine development, approval and global rollout look like, as discussed in the relevant policy and research communities, on the eve of the pandemic? How did these differ from the paths taken during COVID-19?

The development, manufacturing and distribution of COVID-19 vaccines represents a complex process where multiple strands of information, expertise and knowledge intersect. For example, it is necessary to bear in mind there was a background of scientific expertise and technological capability that resulted from the prior years and decades of vaccine R&D (particularly that concerning mRNA vaccines) that made rapid vaccine development after the COVID-19 outbreak possible ( Dolgin 2021). Shorter term factors, such as early predictions of how likely a successful vaccine development was, and how long it was likely to take, should also be considered. This information formed the basis of which decision-makers took early decisions surrounding vaccine investment and advance purchase agreements (a strategy to provide upfront financing for COVID-19 vaccines to accelerate their development and availability) ( Medicines Law & Policy 2021).

Information about the various available vaccines, such as expected effectiveness and potential side-effects, informed decision-making on vaccine eligibility, while waning effectiveness gave rise to the booster-debate. Vaccine mis- and disinformation has proven a great obstacle in ensuring maximum vaccine uptake and has received increasing attention, including in public communication efforts as discussed below.

**Our current knowledge base on this aspect is high, but we still invite people who could provide further insights into the role of information with regards to vaccines—especially on matters of manufacturing and distribution logistics—to share their insights with us.**

### Decision making

1. What decisions were made with regards to national vaccination plans? How and why did countries decide to enter into advance purchase agreements? How were vaccination strategies, *e.g.* age group and risk group prioritisation, decided?
2. How were decisions about global distribution of vaccines made? How were decisions to join or not join COVAX made?
3. How did vaccine developers decide on production targets? How did vaccine developers choose which countries to supply first?

A wide range of decisions had to be made with regards to vaccines, including about early investment and advance purchase agreements, vaccine eligibility and vaccination strategies. More recently, debates have arisen over what rights (of travel, of social mixing, for example) should be associated with vaccination status. On the global level, vaccine inequity has led to calls for more vaccine donations to the Global South. Pledges that have been made so far, such as the G7-pledge, which is still in the implementation phase, have been criticised by campaigners as “too small, too slow and too narrow” ( Wintour 2021; Financial Times 2021). The prospect of mandatory licensing, which might be expected to enable local production in a greater number of countries, is still insufficiently clear. One of the points of interest is the role of scientific experts in vaccine decision-making, as well as what underpins the varying vaccination strategies in different countries.

**Our current knowledge base on this aspect is limited, so we invite people who could provide further insights into the role of decision-making with regards to vaccines to share their insights with us.**

### Infrastructure/implementation

1. Were countries able to roll out vaccines as planned? What were the most common implementation hurdles?
2. What were common challenges in vaccine approval, production, and logistics (transport, storage, and point-of-care delivery)?

There were significant logistical challenges associated with the implementation of vaccination campaigns. Countries had to set up an entire infrastructure ranging from vaccine storage and transportation to vaccine administration at local sites. The challenges were compounded by the strict storage requirements of some of the vaccines, such as the Pfizer vaccine which initially needed to be stored at −70C, requiring a cold supply chain ( Pfizer 2021). The empirical analysis will assess whether countries had the capacities to effectively roll out vaccines and what some of the most common implementation hurdles were.

**Our current knowledge base on this aspect is medium, so we invite people who could provide further insights into the role of infrastructure/implementation with regard to vaccines to share their insights with us.**

### Communication

Communication about vaccines is a crucial pillar in the vaccination effort, to ensure the availability of reliable information about vaccines and thus, it is hoped, increase public confidence in the vaccination campaign. As with most communication, and scientific communication in particular, vaccine communication is multifaceted, as it aims to convey information with regards to the effectiveness and safety of different types of vaccines, practical information (who is eligible? Where can one get the vaccine? *etc.*), and it aims to address mis- and disinformation. The issues raised above with regard to uncertainty and the communication of uncertainty are only amplified. The latter can only effectively be done with an understanding of where these types of false information come from, through reaching out to sceptics and marginalised groups, and through rebuilding trust from the bottom up, such as through trusted community leaders ( US Centers for Disease Control and Prevention 2022).

**Our current knowledge base on this aspect is high, but we still invite people who could provide further insights into the role of communication with regards to vaccines to share their insights with us.**

### Questions

The following is a (non-exhaustive) list of some of the key “open questions” that we have identified to date for this cluster of issues. These are indicative of the ongoing research agenda guiding this phase of our project, and we would be particularly keen to speak with practitioners and experts that can shed further light on answers to some or all of these questions:

1. How have different countries approached vaccine development, approval, purchase, distribution, and communication?
2. Which strategies led to high vaccination rates?
3. What actions impacted other countries’ ability to obtain or distribute vaccines?
4. Which vaccination strategies should be followed, now and in the future? How can we best define vaccine equity and how can it be ensured around the world?

Echoing prior sections of this Research Agenda, we restate here that we aim to answer two fundamental questions in relation to vaccines:

***What would an adequate—or even, an ideal—vaccine infrastructure look like, and what strategy for the development, testing, manufacture, and distribution of COVID-19 vaccines would have most significantly altered the trajectory of the pandemic, and significantly reduced the global loss of life, injury, and harm?***

***What can we learn from the successes and failures of national, regional, and global efforts to develop, test, manufacture and distribute COVID-19 vaccinations to better prepare us to respond effectively to other categories of Global Catastrophic Risk?***

## Cluster #4: non-pharmaceutical interventions

### Background

Non-pharmaceutical interventions (NPIs) are, as defined by the US Centers for Disease Control and Prevention (2020), “actions, apart from getting vaccinated and taking medicine, that people and communities can take to help slow the spread of illnesses like pandemic influenza (flu) (…), also known as community mitigation strategies.” NPIs have been a key instrument in the pandemic response. While they were part of the early response (cluster 2), cluster 4 looks into the use of NPIs during the stage where the virus was widely circulating, so beyond the early action stage. During this period, new information regarding the effectiveness of these measures came in, while compliance with measures became more uneven across the world. This was due to phenomena such as lockdown fatigue and the politicisation of adhering to measures such as mask wearing in certain countries. Other factors, such as populations that are largely reliant on day-labour or non-provision of social goods such as sickness pay, have also influenced the ability and willingness of populations to adhere to these measures. In a number of instances, civil society and grassroots organisations have become involved in cases of low state capacity, aiding communities during the pandemic and fostering broader community resilience.

### Information

1. What information was available about the effectiveness of NPIs on the eve of the pandemic?
2. What information was collected during the pandemic on the effectiveness of NPIs?
3. What information was collected during the pandemic on factors affecting compliance with NPIs?

While on the one hand it is important to assess what information became available about the effectiveness of NPIs during the pandemic, it is also needed to zoom in further on what made these NPIs work. For instance, is financial support necessary to make lockdowns and quarantining effective? And what informs lockdown fatigue and how can countries best address this challenge? How did information on these factors subsequently shape decision making?

Models have been built to estimate how many people would have come into contact with SARS-CoV-2 in the absence of public health measures, including NPIs, and they can therefore give us an indication of how effective these measures were and continue to be. Obviously, there is uncertainty in these models. Yet, they provide us with useful insights in terms of counterfactuals and the effectiveness of adopted measures against COVID-19.

**Our current knowledge base on this aspect is medium, so we invite people who could provide further insights into the role of information with regard to NPIs to share their insights with us.**

### Decision-making

1. Which NPIs did decision-makers opt for? Why?
2. What would an ideal timeline with regards to NPIs have looked like?
3. What local/civil society decisions were made and how did they affect the pandemic response?
4. What was the role of experts in NPI decision making?

As noted in the early action cluster, at the start of the COVID-19 pandemic, various NPIs were introduced in many countries, such as social distancing, the ban of mass gatherings, the closure of schools and offices, and eventually, the introduction of full-scale lockdowns. These measures have been generally quite effective in slowing the spread of SARS-CoV-2, as has been demonstrated by various studies and models ( Thu *et al.* 2021; Future of Humanity Institute 2022). Decision-making on NPIs has generally been contested and hotly debated. Early on in the pandemic, public consensus carried the day in many countries; though this was far from universal. Even in countries where NPI measures were widely accepted and adhered to in the early months of the pandemic, measures such as the cancellation of public events and mask wearing became increasingly contested as the pandemic dragged on, lockdown fatigue set in, and the financial impacts became increasingly clear and difficult to bear.

Mask-wearing has become a political issue in various countries, such as in the United States, where it acts as a fault line between Democrats and Republicans ( Aratani 2020). The role of scientific experts in decision-making processes is also a crucial factor, as many countries institutionalised scientific advisory bodies that provided governments with needed scientific and public health expertise in the pandemic response ( Colman *et al.* 2021). However, the relationship between politicians and scientific experts is a complex one. Politicians generally face a variety of political and societal pressures that in some cases can go directly against the advice of scientific experts, which then potentially adversely affects pandemic decision making. At the same time, experts do not always agree, and there can be contestation between experts and expertise coming from different specialisms. It is important to further elucidate these dynamics to better understand public decisions taken in the context of the COVID-19 pandemic.

In many countries, local civil-society initiatives were set up to help with the pandemic response, supporting people to self-isolate, making masks etc. In some cases, these initiatives were set up in a context of low state capacity, filling important provision gaps. Women-led grassroots groups have often been at the forefront of these initiatives, improving hand washing facilities, setting up community kitchens, and raising awareness about COVID-19, to mention just a few examples ( Sverdlik 2021). It will be important to understand what differences these local, societal responses made in the unfolding of the pandemic.

**Our current knowledge base on this aspect is medium, so we invite people who could provide further insights into the role of decision-making with regard to NPIs to share their insights with us.**

### Infrastructure/implementation

1. What factors contributed to countries’ ability or inability to implement NPIs? Was implementation and enforcement uniform across society? If not, why not?
2. What aspects of infrastructure or details of implementation contributed to public compliance with NPIs?
3. How did local civil-society and community groups organise and deliver support for NPIs? Did they rely on any pre-existing infrastructure, either state-provided or grassroots?

The ability of states to implement NPIs, which relates to their “implementation capacity”, has also played a role in their ability to control the spread of the virus. Especially large states, such as the USA, Brazil, and Russia, found it difficult to control the spread of SARS-CoV-2, partly because of their big populations and high population density in their main cities ( Wong and Li 2020). While some of these states are believed to have ‘high state capacity’, the spread of SARS-CoV-2 was at times so fast that health systems in these countries collapsed or came close to collapsing, as happened to New York City in Spring 2020 ( Armstrong *et al*. 2020). Developing countries, on the other hand, have often lacked the capacity to roll out public health measures in the way that other countries could. This often has to do with years of underinvestment in public health, limited fiscal space, and limited state capacity in general ( UN 2022; Serikbayeva *et al*. 2021). This reflects the uneven impact the pandemic has had globally, which has also been visible on the national level in, for instance, its disproportionate impact on minority groups, such as on BAME communities in the UK ( UK House of Commons Women and Equalities Committee 2020).

This brings us to the issue of compliance with NPIs. Compliance with NPI measures varied between countries and regions even in the early months of the pandemic, however, this compliance also decreased to various extents as the COVID-19 pandemic dragged on ( Six *et al*. 2021, Liu *et al*. 2021). There is some evidence to suggest that compliance may correlate with public fear of the virus. The logic here is that when SARS-CoV-2 was “new”, people tended to fear it, and complied to a large extent with NPIs. Inversely, when the novelty of the virus dissipated, people became gradually less afraid, and compliance decreased accordingly ( Harper *et al.* 2021). Compliance also became less steady through politicisation of some of the public health measures, such as mask wearing and bans on mass gatherings. Especially in Western countries, some of the public health measures were increasingly seen as incompatible with liberal democratic freedoms and resistance against them grew steadily.

As a final point, we have seen innovation with regards to NPIs. Masks soon became a staple of the pandemic response, and allowed for, for instance, safe(r) travel by public transport. Old and trusted test and trace systems were revived through the development of contact tracing apps. “Work from home orders” also worked surprisingly well, particularly in the developed world, due to developments in delivery infrastructures and videoconferencing, and would not have been imaginable on this scale 20 years ago. Other innovations were “hybrid” and “intelligent lockdowns”, which involved closing some public and private places while keeping others open, all on the basis of live monitoring of the public health situation ( de Haas *et al*. 2020; Tullis 2020; de Voogd 2020). This allowed the avoidance of stark trade-offs between full-scale lockdowns and full societal openness. More recently, vaccine passports have been introduced to allow reopening of many societies ( European Commission 2022).

**Our current knowledge base on this aspect is moderate, so we invite people who could provide further insights into the role of infrastructure/implementation with regard to NPIs to share their insights with us.**

### Communication

1. How were NPIs communicated to the public?
2. How did experts and governments communicate in response to resistance to NPIs, e.g. objections to wearing masks or lockdown resistance?
3. What new forms of communication emerge during the pandemic, at the local, national and international level? What new information needs were these new forms addressing, and how well did they perform?

Public communication on NPIs was mostly conducted through press conferences. These kept the general public informed about the applicable measures and provided their rationale. As was the case with vaccines, there was a need to address mis- and disinformation as well. The UK ran the TV-campaign “Stop the Spread” with the WHO in May and June 2020 to raise awareness of the volume of misinformation around COVID-19 and to encourage people to double check information ( WHO 2021). As noted, the task of communication evolved over the course of the pandemic, from crisis communication to a sustained effort that responded to changes in the epidemiological situation, and to emerging issues, such as mis- and disinformation and NPI fatigue surrounding masks and lockdowns.

During the later phases of the pandemic, public communication also faced new challenges: it effectively had to convince the public that countries were still in “crisis mode” amidst growing lockdown fatigue and increasing doubts and resistance against public health measures. It is important to acknowledge and explore the relationship between the nature of certain NPIs and the types of societies they are introduced in, as this relationship touches on various important issues, including compliance, public trust, and the ability of politicians and policymakers to control the spread of SARS-CoV-2.

**Our current knowledge base on this aspect is medium, so we invite people who could provide further insights into the role of communication with regard to NPIs to share their insights with us.**

### Questions

The following is a (non-exhaustive) list of some of the key “open questions” that we have identified to date for this cluster of issues. These are indicative of the ongoing research agenda guiding this phase of our project, and we would be particularly keen to speak with practitioners and experts that can shed further light on answers to some or all of these questions:

1. Which models are most useful in determining the effectiveness of NPIs and assessing counterfactuals and how can these inform present and future pandemic responses?
2. How can one best analyse decision-making processes in the context of the pandemic and the role of scientific experts therein?
3. What are the determinants of compliance with NPIs and how can people best be motivated to comply with measures aimed to control the spread of SARS-CoV-2? To what extent can (non-medical) innovation and technology guide us out of the pandemic?
4. And what does the role of technology and innovation in the pandemic response tell us about their (potential) role in other crises, such as those involving climate change and the loss of biodiversity?

Once again, our aim here is to answer to overarching questions in relation to NPIs:

***What types of NPIs, accompanied by what provision of resources and which communication strategies, would have most significantly altered the trajectory of the pandemic, and significantly reduced the global loss of life, injury, and harm?***

***What can we learn from the successes and failures of national, regional, and global efforts to mitigate or control the COVID-19 pandemic through NPIs in order to better prepare us to respond effectively to other categories of Global Catastrophic Risk?***

## Conclusion: lessons from COVID-19 research agenda, the way forward

The “Lessons from COVID-19” project aims to analyse responses to the COVID-19 pandemic. It aims to draw broader lessons not only for future pandemics, but also for other global catastrophic risks (GCRs), such as extreme climate change and possible catastrophic accidents involving novel technologies. The project will identify and assess key inflection points during the pandemic, when decisions or the lack thereof significantly altered the course of the pandemic, and moved the death toll up or down. Counterfactual analysis will be employed to get a sense of how the trajectory of the pandemic could have been changed for the better based on a different set of interventions in the pandemic response.

Based on our initial survey of secondary sources, a set of early-stage interviews, and our preliminary observations more broadly, efforts to contain the pandemic seem to have effectively failed in the first few months after the outbreak of COVID-19, especially when set against a background of notionally high information availability and preparedness (as compared with other GCRs). In particular, January and February 2020 were lost months, in which early action was lacking in many parts of the world and the opportunity to contain the virus was missed. Complacency and a “wait-and-see” approach gave COVID-19 the chance to transform from an outbreak of concern to a pandemic that could no longer be stopped in its tracks. On top of that, few countries truly had pandemic preparedness in order, a key factor that needs to be addressed to ensure more timely and effective pandemic responses in the future.

This research agenda has formulated key questions with regards to the pandemic response, based on four broad “clusters”, ranging from pandemic preparedness to non-pharmaceutical interventions. Key questions focus on the usefulness and effectiveness of pandemic preparedness plans, the lack of early action and containment measures, vaccine strategies and vaccine equity and the role of science and technology in managing catastrophic crises, such as the COVID-19 pandemic. Crucially, we focus on the decision making around these themes.

We are interested in forging partnerships and collaborations with organisations, experts, and individuals that have expertise in pandemic risk and/or are/were involved with the pandemic response. We believe national and international partnerships are key to learning the right lessons from a pandemic that has affected us all and in translating these lessons into public policies that will better prepare us for present and future pandemics, and catastrophic and existential risks more broadly.

## Data availability

There are no data associated with this article.

## Footnotes

^1^
There are inherent difficulties with estimating COVID-19 death tolls. For a comprehensive and thorough discussion of this challenge, see Adam (2022).

^2^
The Independent Panel for Pandemic Preparedness & Response was formed following a request from the World Health Assembly to the Director-General of WHO in May 2020 to initiate “an impartial, independent, and comprehensive review of the international health response to COVID-19 and of experiences gained and lessons learned from that, and to make recommendations to improve capacities for the future” (Independent Panel 2021, 8). The Panel initiated its work in September 2020 and reported in May 2021. For more information on the Panel’s work, see https://theindependentpanel.org/.

^3^
We acknowledge this is a contested boundary. It is still a useful cut-off point though as the summer/early autumn of 2020 represented a transition period in which many countries had experienced a first wave- often followed by a period in which the pandemic receded to some extent. As more information on the effectiveness of the various public health measures became steadily available during this period of time, countries transited from the early action phase to a sustained pandemic management phase.
