# Supplementary material for: Lessons from COVID-19 for GCR governance: a research agenda
Source: F1000Res. 2024 Jan 3;11:514. Originally published 2022 May 12. [Version 2] doi: 10.12688/f1000research.111331.2 (PMC10904943; doi:10.12688/f1000research.111331.2)
Supplement: Peer review history

# Reviewer report: Reviewer response for version 2

**DOI:** 10.5256/f1000research.160460.r233758

**Published:** 2024-02-15

**Authors:** Ortwin Renn

**Affiliations:** 1 Institute for Advanced Sustainability Studies (IASS), Potsdam, Germany

I appreciate that the authors honored most of the critical remarks that the two reviewers, including me, have made. The authors made some major changes and provided more evidence for what they propose. They also made some corrections and additions concerning lessons for future research and application. I still feel that the paper has many redundancies due to the identical substructure in each phase description but this is tolerable given that this substructure also provides better opportunities for a comparative view.

 Overall I feel that the paper is in a good shape.

Is the topic of the opinion article discussed accurately in the context of the current literature?

Yes

Are arguments sufficiently supported by evidence from the published literature?

No

Are all factual statements correct and adequately supported by citations?

No

Are the conclusions drawn balanced and justified on the basis of the presented arguments?

Partly

Reviewer Expertise:

Risk governance

I confirm that I have read this submission and believe that I have an appropriate level of expertise to confirm that it is of an acceptable scientific standard.

# Reviewer report: Reviewer response for version 1

**DOI:** 10.5256/f1000research.123041.r203305

**Published:** 2023-09-22

**Authors:** Ortwin Renn

**Affiliations:** 1 Institute for Advanced Sustainability Studies (IASS), Potsdam, Germany

Review

 The article provides a metanalysis of the management and governance approaches to deal with the Covid-19 crisis worldwide. The paper defines four broad clusters: pandemic preparedness, early action (from December 2019 to summer/early autumn 2020), vaccines and non-pharmaceutical interventions (NPIs). Within each of these cluster the paper addresses four crucial aspects: information, decision making, infrastructure/implementation and communications. The main objective for this review is to learn from the experiences with COVID 19 in order to be better prepared for other global catastrophic risks.

 The paper summarizes what the authors re-construct as major policy responses during the four phases of the crisis. It also lists potential drivers and reasons for good or bad performance and suggests some lessons for how to improve emergency preparedness and governance for future pandemics but also for other major disasters.

 Each section of the paper ends with a subjective assessment of what the authors believe is their level of knowledge and a series of question for other professionals to ship in their own evidence and knowledge. The lessons for other disasters categories are not explicitly articulated but left open for further input.

 In my view, the endeavor to collect and categorize the experiences from managing and governing the pandemics is extremely ambitious but certainly worth while pursuing. The main challenges here are:

1. Very different performance profiles in different countries (In terms of policies, institutional responsibilities, compliance rates, death toll, health system performance, etc.)
2. Major differences in vulnerability (age distribution, spatial density, status of health systems)
3. Large variety of institutional and legal systems in the countries affected by the pandemic,
4. Different roles of private and public institutions in different countries
5. Large variation in individual compliance, protective actions, and behavioral responses.

In light of these challenges, I find it almost impossible to draw general conclusions about what deficits were encountered and what went wrong on a global perspective. One might cluster countries with similar political culture and institutional settings or select particularly interesting examples such as US, Brazil, New Zealand, Korea, Sweden, Italy and Tanzania.

 A second problem with the paper is the reliance on expert judgement rather than empirical evidence. I am fully aware that there is an abundance of empirical evidence that is hard to digest and ever harder to summarize. This may be an interesting case for using AI systems. Even if other investigators have been asked to contribute, it is unclear whether the authors like to collect more evidence or more expert judgements. It would be good to specify what input in what from the authors expect from the rest of the community. The references in the paper right now represent only a small fraction even of the comparative empirical research and would need to be substantially enhanced if the claim for a comprehensive review is sustained. Alternatively, the authors could systematically collect professional judgments but, in this case, it would be good to use a more organized method such as Delphi or Consensus Conferencing.

 Finally, I would be skeptical about the potential to draw conclusions from the pandemic for other applications of global catastrophes. Other than the normal generalities (be better prepared, have emergency management teams ready before disaster strikes, engage in effective communication, etc.) many of the mentioned global disasters such as global climate change or being hit by a large meteorite, require management and governance responses that differ al lot from the context of pandemics. Unless the authors provide some good evidence or arguments that show the transferability of lessons from COVID to be meaningfully applied to other disasters, I am not convinced.

 Overall, I endorse the experiment to organize a collective effort to synthesize the experiences from the COVID crisis and start a mutual learning process by asking for input from the scientific and policy communities worldwide. However, I would suggest a far more structured approach, a clearer distinction between countries and political cultures, and more specific set of questions and input categories for potential contributors and a more differentiated perspective when applying insights from the pandemic to other disaster areas.

Is the topic of the opinion article discussed accurately in the context of the current literature?

Yes

Are arguments sufficiently supported by evidence from the published literature?

No

Are all factual statements correct and adequately supported by citations?

No

Are the conclusions drawn balanced and justified on the basis of the presented arguments?

Partly

Reviewer Expertise:

Sociology and Risk Governamnce

I confirm that I have read this submission and believe that I have an appropriate level of expertise to confirm that it is of an acceptable scientific standard, however I have significant reservations, as outlined above.

## References

[1] : COVID-19 risk governance: drivers, responses and lessons to be learned. *Journal of Risk Research* .2020;23(7-8) : 10.1080/13669877.2020.1760332 1073-1082 10.1080/13669877.2020.1760332

# Response

**Published:** 2023-12-20

**Authors:** Jochem Rietveld

**Affiliations:** University of Cambridge, UK

First of all, we’d like to express our sincere thanks for the time and effort invested in writing this review. This is very much appreciated.

 Our response will now proceed in a point-by-point format

 Re- input from the rest of the community:

 We agree further specification in this area would be helpful. With your suggestions in mind, we have added a paragraph to the introduction in which we outline two main routes for implementation of our research agenda: an ‘expert judgments route’ through use of the Delphi method and/or a ‘empirical data route’ through systematic data collection by a collective of researchers.

 Re-very different performance profiles in different countries:

 We recognise this challenge. We hope and expect to find patterns in response and challenges experienced by many countries. These patterns and challenges can be illustrated by using country examples. We do not aim to provide any conclusive accounts about specific countries or clusters of countries. While there is significant merit in such an approach, this is beyond the scope of our endeavour. However, if there is an interest among researchers in taking the above approach, we would of course encourage them to do so

 Re- management and governance responses vis-a-vis different global disasters:

 We recognise that specific global disasters will require tailored management and governance responses. It is clear that certain lessons from the COVID-19 pandemic will be limited in scope and may only be transferable to future pandemics of a similar viral agent. One can think of the patterns of spread of SARS-CoV-2 and the lessons that have been learnt in mitigating against this spread, through, for instance, social distancing and mask wearing. These are lessons that have little relevance for, say, meeting the challenge of climate change and rapid AI development and deployment. We believe, however, that there are also transferable lessons to be identified and learnt.

 The reviewer has a point when he calls these the usual ‘generalities, such as ‘be prepared, communicate effectively’ etc. However, while these ‘generalities’ may have been highlighted in the academic literature and national and international and policy circles for years, including before the COVID-19 pandemic, it is evident that the pandemic has exposed that these insights have been insufficiently implemented in policy practice, both at the national, regional, and global level.

 The pandemic has also offered a significant case to assess how all the insights in governance, communication, and leadership have played out in practice. This has led to new insights and perspectives, as well as the revision of ‘established wisdoms’ in public health, such as those held around dominant ways of viral spread. Misinformation and disinformation, while not new, have had an outsized influence on the pandemic response on a scale not seen in crises in recent years and governments struggled to deal with this challenge. Overall the pandemic has demonstrated that while many of the ‘usual generalities’ such as ‘be prepared’ and ‘communicate effectively’ continue to hold, these also require to be continuously updated and redefined in the face of new developments and challenges.

# Reviewer report: Reviewer response for version 1

**DOI:** 10.5256/f1000research.123041.r156391

**Published:** 2023-01-04

**Authors:** Stephen R. Hanney

**Affiliations:** 1 Health Economics Research Group, Brunel University London, Uxbridge, UK

This generally well-written, ambitious opinion paper provides a research agenda or structure to study the response to the COVID-19 pandemic, and learn lessons from a Global Catastrophic Risk (GCR) perspective. Using this structure it outlines the team’s current knowledge base on a series of issues, and asks if readers can provide insights on specific points.

 The excellent aims of the study, for which this paper provides the research agenda, include investigating key decisions (or failures to decide) that altered the course of the pandemic, and then using the lessons to improve pandemic preparedness in future. The study also seeks to ask how the lessons could be transferred to other areas of potential GCR management such as extreme climate change and radical loss of biodiversity. The study aims to identify the key moments, or *“inflection points”*, through examining what are called four broad clusters: pandemic preparedness, early action (from December 2019 to summer/early autumn 2020), vaccines and non-pharmaceutical interventions (NPIs). Four aspects are listed that are common across all four clusters and can be used to assess the possible inflection points in each cluster. These aspects are: information, decision making, infrastructure/implementation and communications.

 The research agenda identifies different and specific key questions for each of the four aspects in turn, in each of the four clusters. The paper provides some text addressing the questions and at the end of each aspect indicates whether the team’s knowledge base is high, moderate or low but, irrespective of that, invites readers to provide further insights. Additionally, at the end of each cluster there is a concluding section listing some of the open questions that, the authors say, *“are indicative of the ongoing research agenda guiding this phase of our project”. *They say they would particularly like to speak to experts and practitioners who can shed light on those questions.

 The many strengths of the paper suggest that the research agenda is likely to provide a sound basis for a substantial analysis of key parts of the pandemic, and the identification of useful lessons. For example, the first main section is on Cluster #1, pandemic preparedness, and it starts with the important observation from a World Health Organization (WHO) report that pandemic preparedness should be defined as *“a continuous process”.* Using this as the definition helps focus the analysis on where the shortcomings emerged, and how notions of preparedness have now started to evolve.

 There are, however, considerable challenges facing the task of constructing and presenting a research agenda in this field. An enormous number of studies have already been conducted on diverse aspects of the COVID-19 pandemic, and thousands of papers have already been published. Therefore, constructing a new research agenda that adequately reflects, incorporates and builds on the existing state of knowledge requires considerable effort and thought. As noted, the authors ingeniously attempt to alleviate this challenge, and indeed bolster the pool of evidence for them to analyse, by inviting readers at the various points to share their own insights. In this way the authors are themselves, at times, indicating that they are aware that some arguments should be supported by additional evidence from the published literature, and that they are actively seeking such evidence for their ongoing study.

 Being invited to review this paper some six months after its publication in May 2022, itself presents challenges and opportunities because some of the inevitably large number of suggestions about further insights that could be made, and the additional evidence, come from the stream of publications that continued through that six months. In particular, there were other comprehensive studies that attempted to provide lessons from the pandemic based on wide-ranging analysis. Some of these drew on and collated evidence from hundreds of earlier publications (The Lancet Commission, 2022; Hanney et al, 2022). While, of course, these collations published after May 2022 could not have been included in the paper under review, many of the insights in them are drawn from analysis of earlier publications. Having acknowledged this point, the overlaps mean it is not helpful for this review to be divided into insights that had been available before May 2022, and those now available. Instead, from here on this review will be divided into two main parts (with one common reference list):

**A) Suggestions made as a reviewer of points where possible amendments should be seriously considered in future editions of the paper.**

**B) Comments made as a reader in response to the authors’ requests for additional insights to consider for possible inclusion in the study, and possibly in any future editions of the paper, as the authors see fit.** Many of these comments are based on analysis included in the two recent collations of evidence described above, and cite papers whose contributions are usually described at greater length in one or both of those two collations. (It might be worth noting, however, that along with much excellent analysis, there are some points in the report of the Lancet Commission 2022 on lessons for the future from the COVID-19 pandemic that do not seem consistent with the evidence published elsewhere).

***A) Suggestions as a reviewer of points where possible amendments should seriously be considered in future editions of the paper.***

1. Introduction, and repeated elsewhere. As noted, one of the aims of the paper is to identify lessons that could be transferred to other areas of potential GCR management such as extreme climate change and radical loss of biodiversity. This is a laudable aim, but I think the paper would benefit from a somewhat further explanation of how *“extreme climate change”* and *“radical loss of biodiversity”* are sufficiently parallel to the rapid emergence of the pandemic to allow many lessons to be drawn.  The authors themselves claim that January and February 2020 *“were lost months, in which early action was lacking in many parts of the world and the opportunity to contain the virus was missed”* (pp.17/18). That seems correct, but also underlines a key difference of timescale with the other GCRs above where the crises seem to be building up over many years.  Perhaps this distinction could also additionally be considered as a possible limitation towards the end of the article?
2. Introduction. While the Introduction generally sets the paper up well, I think it might be easier for the reader to absorb the key point that the clusters will be used to organise the rest of paper if some of the points were presented slightly differently here. In the Introduction there is a numbered list of four questions that the study is asking about the decisions taken in the pandemic and counterfactuals about what could have been done differently. Then there is a very brief paragraph in which the four clusters of issues to be studied are included in a brief sentence with no numbering of the points. Then the four aspects to be considered in each cluster are described, with numbers attached to each one. However, the remainder of the full paper is organised according to the clusters, with each one of the subsequent main sections headed with the number and name of the relevant cluster. Therefore, perhaps it would be helpful in the Introduction if it was the clusters that were presented more prominently and in a numbered list.
3. Cluster #1: pandemic preparedness/Cluster #2: early action. In Cluster #2 the paper highlights decision making in the early phase in China and other Asian countries. Below, however, I outline how there were also important examples of early action in Australia and New Zealand, and these might suggest it could be better to consider referring (alternatively or also) to WHO’s Western Pacific Region as the location for examples of early action? Useful evidence from Australia about early action is linked to pandemic preparedness and research. Coordinated research preparedness had occurred through the 13 organizations who were members of the Australian Partnership for Preparedness Research on Infectious Disease Emergencies (APPRISE) that was created in 2016. It responded to COVID-19 by activating a pre-planned research platform as early as 13 ^th^ January 2020 (National Health and Medical Research Council, 2020). In New South Wales (NSW) the government’s response built on previous pandemic planning and as early as 21 ^st^ January 2020 NSW Health opened its Public Health Emergency Operations Centre. This was ready if, and when, necessary, *“to coordinate case finding, contact tracing, outbreak control, communications, and other preventive actions”* (McAnulty & Ward, 2020). There was also effective early action in New Zealand shortly after the first cases were reported, although in this instance it was not so much a result of earlier pandemic preparedness (Geoghegan et al, 2021). There are also wider accounts of evidence from Australasia and countries such as Germany that analyse the valuable role of health research systems in working closely with politicians in developing and using evidence for early and effective action, including to introduce NPIs (Hanney et al, 2022 - see especially pp. 23-28). (This issue is further discussed in point 5 below). 
4. Cluster #3: Vaccines. On pp.12/13 the paper rightly refers to the remarkable speed of the vaccine development by multiple companies, particularly for the mRNA vaccines, and how this technology was based on many years of prior work by expert scientists. While all this is correct and relevant, I think the years of work by scientists at Oxford University should also be featured as their rapid COVID vaccine development was similarly based on their earlier work that had led to the development of a vaccine platform that was available when COVID-19 arrived. According to Sarah Gilbert, lead developer of the vaccine, the team had been “ *thinking about an appropriate response to Disease X; how could we mobilise and focus our resources to go more quickly than we had ever gone before. And then Disease X arrived”* (Lane, 2020; Gilbert & Green, 2021).
5. Cluster #4: NPIs (p.14-17) in relation to Cluster #2: early action (p.9-11).  The paper states *“cluster 4 looks into the use of NPIs during the stage where the virus was widely circulating, so beyond the early action stage”*. The paper also acknowledges NPIs were part of the early action, but, as noted, above, the effective early introductions of NPIs in Australia and New Zealand etc had considerable success, and while selected NPIs (such as border controls) were continued after the early phase, the virus was still not widely circulating in those countries. Therefore perhaps in that context, and to enhance understanding about the effective use of NPIs, it would be valuable to consider going further and applying some of the Cluster #4 questions about NPIs specifically to evidence from those countries.  Examples include the actions by the NSW Health that brought researchers in to the department to co-produce evidence on NPIs and other policies (Campbell et al, 2021). Similarly, in New Zealand, the experts played a key role in informing action, and research teams with links to the Ministry of Health produced valuable evidence about how highly effective the NPIs had been, and also could potentially provide useful evidence in relation to the following question in Cluster #4, Decision-making: *“What would an ideal timeline with regards to NPIs have looked like”* (Jefferies et al, 2020; Geoghegan et al, 2021). (However, fuller relaxation of NPIs in both countries was followed in 2022 by a large increase in cases, but the high level of vaccination achieved by then meant the cumulative death rate in each country, while considerably increased, was still much lower than in most other countries).

**B) Comments made as a reader in response to the authors’ requests for additional insights to consider for possible inclusion in the study, and possibly in any future editions of the paper, as the authors see fit.**

1. In terms of how far preparedness helped countries respond well to the pandemic, various insights might come from comparing the pandemic performance of countries with the data published in the Global Health Security (GHS) Index in October 2019 about the countries' potential level of health security preparedness (GHS Index, 2019). While the GHS Index went much wider than just pandemic preparedness, and found no country was completely prepared for a major health emergency, various authors raised questions about why the countries ranked first and second on the GHS Index, ie the US and the UK, had a much higher death rate from COVID-19 than many other countries (Nuzzo et al, 2020; Tworek et al, 2020; Farrar, 2021; Hanney et al, 2022; The Lancet Commission, 2022). 
2. The Cluster #2: early action, Information section starts with the question: “ *What information was available about SARS-CoV-2 in the early days and weeks immediately following its discovery?*” A range of important insights about this can be found in the early chapters of the authoritative account by Jeremy Farrar, with Anjana Ahuja, called *Spike: The Virus vs The People. The Inside Story* (Farrar, 2021).
3. In the Cluster #2: early action, Decisions section, particularly where the role of WHO is discussed, further insights might come from drawing on the Lancet Commission Report’s sections on the early response and WHO shortcomings (pp.10-12) (The Lancet Commission, 2022), and the WHO’s response to the report’s criticisms (WHO, 2022).
4. There are insights about the unique research infrastructure existing in the UK at the start of 2020 that, while not specifically related to pandemic preparedness per se, might be relevant for several of the clusters. It is widely agreed that it was the research infrastructure of the National Institute for Health Research (NIHR), embedded across the National Health Service (NHS),  that facilitated the Randomised Evaluation of COVID-19 Therapy (RECOVER) trial’s rapid recruitment and globally leading progress to identify existing drugs that were, or were not, effective therapies for treating COVID-19 (Lane & Fauci, 2020; Pessoa-Amorim et al, 2021; Hanney et al, 2022). 
5. For the Cluster #2: early action, Communications section, additional analyses not only concur with the examples of effectiveness already identified in paper, including the role Germany’s Angela Merkel and New Zealand’s Jacinda Ardern, but also provide further insights. Additional examples of effectiveness are reported from jurisdictions such as Senegal, South Korea and the Canadian province of British, along with an attempt to list common features found in many examples of effective pandemic communication that might be useful if more widely adopted (Tworek et al, 2020). 
6. For Cluster #3: vaccines, there are many additional studies available that can provide further insights on some of the questions asked in the paper. In the information section, one question relates to the nature of proposals being considered in research communities prior to the pandemic for rapid vaccine development, and what actually happened. Here, previous analysis proposed that research on innovations such as drugs and vaccines could be accelerated through approaches including increasing resources, working in parallel, starting or working at risk and improving processes (Hanney et al, 2015). Rapid analysis of the work on developing COVID-19 vaccines suggested examples of all of these approaches could be seen (Hanney et al, 2020). This paper also considered which of the factors it might be possible to replicate in future on a regular basis, and which factors, such as the extreme concentration of resources and avoidance of the usual queues for decisions, might only be possible in a pandemic-type situation. In addition to the improved processes noted above through new vaccine platforms, there were striking examples of working in parallel, and crucially including early manufacture in that (Lurie et al, 2020) along with provision of greatly increased resources, in particular with Operation Warp Speed in the US which combined enormous financial resources with the logistical expertise provided through the Department of Defense (Slaoui & Hepburn, 2020; Koehlmoos et al, 2022) **. **In the Decision making section, the Lancet Commission provides further insights on questions about how decisions were made about the global distribution of vaccines and the COVID-19 Vaccines Global Access Facility (COVAX) (The Lancet Commission, 2022).

Is the topic of the opinion article discussed accurately in the context of the current literature?

Yes

Are arguments sufficiently supported by evidence from the published literature?

Partly

Are all factual statements correct and adequately supported by citations?

Yes

Are the conclusions drawn balanced and justified on the basis of the presented arguments?

Yes

Reviewer Expertise:

The organisation and impact of health research and health research systems.

I confirm that I have read this submission and believe that I have an appropriate level of expertise to confirm that it is of an acceptable scientific standard.

## References

[1] : NSW Health COVID-19 Emergency Response Priority Research program: a case study of rapid translation of research into health decision making. *Public Health Res Pract* .2021;31(4) : 10.17061/phrp3142124 10.17061/phrp3142124 34753169

[2] : Spike: The Virus vs. The People - the Inside Story. *Profile Books* .2021;

[3] : New Zealand's science-led response to the SARS-CoV-2 pandemic. *Nat Immunol* .2021;22(3) : 10.1038/s41590-021-00872-x 262-263 10.1038/s41590-021-00872-x 33627881

[4] : Vaxxers: A Pioneering Moment in Scientific History. *Hodder* .

[5] : Global Health Security Index – Building Collective Action and Accountability. *Global Health Security Index* .2019; Reference source

[6] : Saving millions of lives but some resources squandered: emerging lessons from health research system pandemic achievements and challenges. *Health Res Policy Syst* .2022;20(1) : 10.1186/s12961-022-00883-6 99 10.1186/s12961-022-00883-6 36088365 PMC9464102

[7] : How long does biomedical research take? Studying the time taken between biomedical and health research and its translation into products, policy, and practice. *Health Res Policy Syst* .2015;13: 10.1186/1478-4505-13-1 1 10.1186/1478-4505-13-1 25552353 PMC4297458

[8] : From COVID-19 research to vaccine application: why might it take 17 months not 17 years and what are the wider lessons?. *Health Research Policy and Systems* .2020;18(1) : 10.1186/s12961-020-00571-3 10.1186/s12961-020-00571-3 32513202 PMC7276964

[9] : COVID-19 in New Zealand and the impact of the national response: a descriptive epidemiological study. *The Lancet Public Health* .2020;5(11) : 10.1016/S2468-2667(20)30225-5 e612-e623 10.1016/S2468-2667(20)30225-5 PMC755390333065023

[10] : The collaborative research and service delivery partnership between the United States healthcare system and the U.S. Military Health System during the COVID-19 pandemic. *Health Res Policy Syst* .2022;20(1) : 10.1186/s12961-022-00885-4 81 10.1186/s12961-022-00885-4 35854348 PMC9295105

[11] : Sarah Gilbert: carving a path towards a COVID-19 vaccine. *The Lancet* .2020;395(10232) : 10.1016/S0140-6736(20)30796-0 10.1016/S0140-6736(20)30796-0 PMC716264432305089

[12] : Research in the Context of a Pandemic. *New England Journal of Medicine* .2021;384(8) : 10.1056/NEJMe2024638 755-757 10.1056/NEJMe2024638 PMC738359132678528

[13] : Developing Covid-19 Vaccines at Pandemic Speed. *N Engl J Med* .2020;382(21) : 10.1056/NEJMp2005630 1969-1973 10.1056/NEJMp2005630 32227757

[14] : Suppressing the Epidemic in New South Wales. *New England Journal of Medicine* .2020;382(21) : 10.1056/NEJMc2011592 10.1056/NEJMc2011592 PMC723330432383832

[15] Australian Partnership for Preparedness Research on Infectious Disease Emergencies. *National Health and Medical Research Council (NHMRC)* .2020; Reference source

[16] : Suboptimal US Response to COVID-19 Despite Robust Capabilities and Resources. *JAMA* .2020;324(14) : 10.1001/jama.2020.17395 10.1001/jama.2020.17395 32936215

[17] : Making trials part of good clinical care: lessons from the RECOVERY trial. *Future Healthc J* .2021;8(2) : 10.7861/fhj.2021-0083 e243-e250 10.7861/fhj.2021-0083 34286192 PMC8285150

[18] : Developing Safe and Effective Covid Vaccines - Operation Warp Speed's Strategy and Approach. *N Engl J Med* .2020;383(18) : 10.1056/NEJMp2027405 1701-1703 10.1056/NEJMp2027405 32846056

[19] : The Lancet Commission on lessons for the future from the COVID-19 pandemic. *The Lancet* .2022;400(10359) : 10.1016/S0140-6736(22)01585-9 1224-1280 10.1016/S0140-6736(22)01585-9 PMC953954236115368

[20] : Democratic Health Communications during Covid-19: A RAPID Response. *Vancouver: UBC Centre for the Study of Democratic Institutions* .2020; Reference source

[21] WHO responds to The Lancet COVID-19 Commission. *World Health Organization* .2022; Reference source

# Response

**Published:** 2023-12-20

**Authors:** Jochem Rietveld

**Affiliations:** University of Cambridge, UK

First of all, we’d like to express our sincere thanks for the time and effort invested in writing this review. This is very much appreciated.

 Our response will now proceed in a point-by-point format

 A)

 1. The difference in timescales has now been acknowledged in the introduction. A note has also been added that this may limit crosscutting insights from the pandemic to some other risk areas that seem to operate on different timescales.

 2. The clusters have now been numbered in the introduction, while the four aspects have been given letters instead of numbers, to clarify the article’s structure.

 3. We have now included a mention of the early interventions of Australia and New Zealand in the early action cluster (cluster 2).

 4. The work of the Oxford team that developed a platform to prepare for the arrival of ‘disease X’ has now been acknowledged in the vaccine cluster (cluster 3)

 5. The success of Australia and New Zealand in preventing widespread circulation of the virus through the effective use of NPIs has now been added to the NPI cluster (cluster 4). This includes a note that this makes them particularly interesting cases in this cluster.

 B)

 While no immediate in-text edits have been made in response to the points made under B), we very much welcome these additional insights offered by the reviewer and see these as important starting points for the implementation of our research agenda.
